# Machine learning-based evaluation of spontaneous pain and analgesics from cellular calcium signals in the mouse primary somatosensory cortex using explainable features

**DOI:** 10.3389/fnmol.2024.1356453

**Published:** 2024-02-21

**Authors:** Myeong Seong Bak, Haney Park, Heera Yoon, Geehoon Chung, Hyunjin Shin, Soonho Shin, Tai Wan Kim, Kyungjoon Lee, U. Valentin Nägerl, Sang Jeong Kim, Sun Kwang Kim

**Affiliations:** ^1^Department of Science in Korean Medicine, Graduate School, Kyung Hee University, Seoul, Republic of Korea; ^2^Division of AI and Data Analysis, Neurogrin Inc., Seoul, Republic of Korea; ^3^Division of Preclinical R&D, Neurogrin Inc., Seoul, Republic of Korea; ^4^Department of Physiology, College of Korean Medicine, Kyung Hee University, Seoul, Republic of Korea; ^5^Department of Physiology, Seoul National University College of Medicine, Seoul, Republic of Korea; ^6^Department of Korean Medicine, Graduate School, Kyung Hee University, Seoul, Republic of Korea; ^7^Department of East-West Medicine, Graduate School, Kyung Hee University, Seoul, Republic of Korea; ^8^Interdisciplinary Institute for Neuroscience, CNRS UMR 5297 and University of Bordeaux, Bordeaux, France

**Keywords:** brain cellular calcium, spontaneous pain, machine learning, animal models, explainable features

## Abstract

**Introduction:**

Pain that arises spontaneously is considered more clinically relevant than pain evoked by external stimuli. However, measuring spontaneous pain in animal models in preclinical studies is challenging due to methodological limitations. To address this issue, recently we developed a deep learning (DL) model to assess spontaneous pain using cellular calcium signals of the primary somatosensory cortex (S1) in awake head-fixed mice. However, DL operate like a “black box”, where their decision-making process is not transparent and is difficult to understand, which is especially evident when our DL model classifies different states of pain based on cellular calcium signals. In this study, we introduce a novel machine learning (ML) model that utilizes features that were manually extracted from S1 calcium signals, including the dynamic changes in calcium levels and the cell-to-cell activity correlations.

**Method:**

We focused on observing neural activity patterns in the primary somatosensory cortex (S1) of mice using two-photon calcium imaging after injecting a calcium indicator (GCaMP6s) into the S1 cortex neurons. We extracted features related to the ratio of up and down-regulated cells in calcium activity and the correlation level of activity between cells as input data for the ML model. The ML model was validated using a Leave-One-Subject-Out Cross-Validation approach to distinguish between non-pain, pain, and drug-induced analgesic states.

**Results and discussion:**

The ML model was designed to classify data into three distinct categories: non-pain, pain, and drug-induced analgesic states. Its versatility was demonstrated by successfully classifying different states across various pain models, including inflammatory and neuropathic pain, as well as confirming its utility in identifying the analgesic effects of drugs like ketoprofen, morphine, and the efficacy of magnolin, a candidate analgesic compound. In conclusion, our ML model surpasses the limitations of previous DL approaches by leveraging manually extracted features. This not only clarifies the decision-making process of the ML model but also yields insights into neuronal activity patterns associated with pain, facilitating preclinical studies of analgesics with higher potential for clinical translation.

## 1 Introduction

Pain management, especially for chronic conditions, remains a significant challenge in the medical field (Bushnell et al., 2013). Chronic pain not only disrupts daily life and increases distress but also imposes a substantial socio-economic impact (Annagür et al., 2014, Petrosky et al., 2018). The current landscape of analgesics, which includes a range of medications, presents its own issues. They often fall short in effectively treating chronic pain. In case of narcotic drugs, opioids being one example, they are associated with severe side effects, ranging from addiction to life-threatening conditions. The abuse of them has become a serious public health crisis, highlighting the urgent need for safer and more effective pain management strategies (Hedegaard et al., 2017; Blanco et al., 2020). Given these challenges, there is a pressing demand for innovative approaches to understand and treat chronic pain more effectively.

The challenge in developing pain medicines arises from discrepancies between animal tests and human studies (Mogil and Crager, 2004; Mogil, 2009). In many pain conditions, patients predominantly experience spontaneous pain (Galer et al., 2000; Staud et al., 2007; Latremoliere and Woolf, 2009; Maier et al., 2010; Hanagasi et al., 2011). However, animal studies often focus on pain that is evoked by specific external stimuli. Although spontaneous pain is more problematic than evoked pain in real life (Murai et al., 2016), yet current research methodologies cannot fully capture this spontaneous pain so that numerous studies have focused only on evoked pain. This may overlook critical aspects of spontaneous pain, potentially hindering the development of effective treatments. Aligning research with the actual experiences of patients is of paramount importance in development of appropriate treatments (Rice et al., 2008).

In recent research, chronic pain models have been validated for spontaneous pain through various methods, including the Grimace Scale, Conditioned Place Preference (CPP), and observations of nocifensive behaviors. However, these traditional approaches often lack accuracy and objectivity, particularly in chronic pain scenarios. For instance, chronic pain in animals typically manifests through intermittent signs like flinching, which are sporadic and challenging to measure objectively (Mogil et al., 2010). The effectiveness of the Grimace Scale diminishes in chronic states (Langford et al., 2010; De Rantere et al., 2016), and while CPP provides some insights, it faces challenges in both quantifying pain and distinguishing between analgesic effects and preference itself, limiting its accuracy (King et al., 2009). Additionally, the utility of CPP depends on factors such as the integrity of both the reward and the learning and memory systems, which can restrict its suitability for certain disease models. Recognizing these limitations, our study adopts a machine learning model that analyzes spontaneous pain from calcium imaging data, thus addressing the shortcomings of conventional methods and enhancing the objectivity and reliability of pain assessment in animal models.

Leveraging advancements in computational techniques, ML has become a key tool in pain research, outperforming prior methods in analyzing complex calcium imaging data (Singh et al., 2016; Boissoneault et al., 2017; Lötsch and Ultsch, 2018; Jones et al., 2020). Previously, we developed a DL model for assessing spontaneous pain (Yoon et al., 2022). DL is adept at detecting complex patterns in pain data, often missed by human analysis, thereby offering a more explicit detection of pain. However, DL approach had limitations, particularly in its lack of transparency (Salahuddin et al., 2022), which made it difficult to detect malfunctions of neural circuits. In this study, we adopted machine learning (ML) approach to gain deeper insights into the patterns of S1 calcium signals associated with spontaneous pain, as the ML approaches are anticipated to offer enhanced transparency and intuitive understanding. This is due to the explicit and user-friendly nature of ML, facilitating effective correction of biases and errors within the system (Mi et al., 2021). Therefore, developing a ML model for spontaneous pain assessment could offer several advantages over DL, such as improved transparency for early malfunction detection and enhanced adaptability in modifying features to correct errors.

Despite the considerable advancements ML has brought to pain research, particularly in analyzing complex calcium imaging data, its application in the development of new analgesics introduces another challenge. Binary classification models, which typically label data as either “pain” or “no pain,” may oversimplify the complex nature of pain and condition altered by its treatment (Lötsch and Ultsch, 2018). The challenge arises when trying to identify truly novel analgesics that have not been explored before. Traditional models might not be equipped to recognize or categorize these new entities effectively. Furthermore, any medication, including analgesics, inherently carries multiple side effects (Lamon et al., 2008; Young et al., 2018). An oversimplified model might misinterpret these side effects, leading to potential risks in drug development. A recent study has indicated that multi-class classification systems offer more detailed and accurate diagnostics than binary classification systems (Mi et al., 2021). Consequently, employing a multi-classification method could be more effective in identifying the varied effects of analgesics.

Our primary goal in this study is to offer a more transparent and interpretable method for measuring spontaneous pain in preclinical research. Unlike previous research that predominantly relied on DL with black-box mechanisms (Yoon et al., 2022), we adopt a ML approach that emphasizes manual feature extraction. This methodology aims to provide a clearer understanding of the reasons behind the classification of S1 calcium signals as “pain” or “no pain.” Furthermore, by extending beyond the traditional binary classification and introducing a third-class label, we seek to achieve a more detailed interpretation of neural activity patterns, discerning whether the neural activity pattern induced by candidate drugs mirrors existing analgesics or introduces a new signature.

## 2 Methods

### 2.1 Experimental animals and housing conditions

C57BL/6 male mice were 6 weeks old at the start of the experiments. To reduce stress, they were grouped in batches of two to four. The living conditions maintained a 12-h light/dark rotation, and animals had free access to water and food. The experimental protocols received approval from Kyung Hee University Institutional Animal Care and Use Committee [KHUASP (SE) 22-280], in line with the National Institutes of Health's guidelines.

### 2.2 Experimental models of spontaneous pain

To establish the complete Freund's adjuvant (CFA) model (Yoon et al., 2022), 10 μl of CFA were injected subcutaneously into the plantar surface of the right hind paw. To establish the chemotherapy-induced peripheral neuropathy model (Kim W. et al., 2016), oxaliplatin (6 mg/kg) was treated intraperitoneally. To establish the nerve injury-induced neuropathic pain model, the mice underwent partial sciatic nerve ligation (PSL) surgery (Korah et al., 2022) on the right hind paw. The right sciatic nerve was exposed at the upper thigh of the mouse, and one-third to one-half of the nerve diameter was ligated with a 9-0 suture. To establish Parkinson's disease (PD) model, mice were injected intraperitoneally with 1-methyl-4-phenyl-1,2,3,6-tetrahydropyridine (MPTP, 30 mg/kg) daily for 5 days (Hwang et al., 2019).

In the assessment of the analgesic effects, morphine was administered intraperitoneally to the PSL model at a dose of 10 mg/kg and to the PD model at a dose of 5 mg/kg. For the CFA model, ketoprofen was administered intraperitoneally at a dose of 50 mg/kg. Magnolin was initially administered as a single dose by intraperitoneal injection to the PSL model, followed by twice-daily doses for four consecutive days. The single dose was administered once at 30 mg/kg, and the repeated dose was administered at 30 mg/kg twice a day. All models were administered the analgesics 30 min before behavior tests and imaging.

### 2.3 Behavior tests

The rotarod test was used to assess motor coordination in mice. Mice were placed on a rotating rod that gradually increased in speed from 5 to 40 rpm over a period of 300 seconds. The test was terminated when the mouse fell off the rod. Each mouse was tested three times with a minimum of 5 min between trials.

The von Frey test was used to assess mechanical hypersensitivity in mice. Mice were placed in individual, clear plastic cages with mesh floors. A series of von Frey filaments with bending forces of 2.36, 2.44, 2.83, 3.22, 3.61, 3.84, 4.08, and 4.31 g were applied to the plantar surface of each hind paw using the up-down method. The paw withdrawal threshold, defined as the force at which the mouse consistently withdrew its paw in response to five consecutive filament applications, was determined for each animal (Chaplan et al., 1994).

### 2.4 Immunohistochemistry

At the end of the behavior tests, the mice were deeply anesthetized with isoflurane and fixed in 4% paraformaldehyde (PFA) via the left ventricle. Brains were harvested and fixed in 4% PFA overnight at 4°C and then dehydrated in 30% sucrose solution until the brains sank. The brains were then cut into 40 μm coronal sections containing Substantia Nigra compacta (SNc) using a cryostat (Thermo Fisher Scientific, Waltham, MA, USA). The sections were incubated with 1% hydrogen peroxide for 15 min to eliminate endogenous peroxidase activity, and sections were blocked for 1 h in a solution containing 3% bovine serum albumin and 0.3% Triton X-100. They were activated with anti-tyrosine hydroxylase (TH) antibody (1:1000, Sigma-Aldrich, St. Louis, MO, USA) for 72 h at 4°C. They were activated with biotinylated anti-rabbit IgG (Vector Laboratories, Burlingame, CA, USA) for 1 h and a solution of avidin-biotinylated peroxidase complex (Vectastain Elite ABC kit, Vector Laboratories, Burlingame, CA, USA) for 1 h. Sections were stained with 3,3′-diaminobenzidine (DAB, DAB Substrate Kit, Peroxidase, Vector Laboratories, Burlingame, CA, USA) for approximately 40 seconds. The stained sections were attached to aminosilane-coated slide glasses and dehydrated in 70, 80, 90, and 100% ethanol. Slides were blotted with xylene and mounted using permount. The histological images of SNc were obtained using a bright-field microscope (Nikon, Tokyo, Japan). The number of dopaminergic neurons was counted using a deep learning-based auto detection algorithm, and manually verified (Kim D. et al., 2023).

### 2.5 Procedure for two-photon calcium imaging

All surgical operations were conducted under combined anesthesia with Zoletil (30 mg/kg) and xylazine (10 mg/kg). A cranial window, measuring 2 × 2 mm, was crafted above the left S1 cortical hind paw region (positioned laterally by 1.5 mm and posteriorly by 0.5 mm from Bregma) to facilitate longitudinal calcium observations. We utilized a surgical blade (#11) for this. The S1 was injected with the Adeno-associated virus showcasing GCaMP6s (from the University of Pennsylvania Gene Therapy Program Vector Core). After the viral injection, a thin cover glass (sourced from Matsunami, Japan) was placed over the cranial window, sealed securely with Vetbond (3M) and dental cement.

Over a period of 2 weeks, the mice underwent acclimatization on a treadmill while their heads were stabilized, spending 40 min daily. The imaging process used a two-photon microscope (FVMPE-RS, Olympus, Tokyo, Japan) with a water immersion objective lens (XLPlan N 25, NA = 1.05, Olympus, Tokyo, Japan). The GCaMP6s indicator was excited by900 nm light provided by a Ti: sapphire laser system (Chameleon, Coherent, USA). Capturing of the imaging frames was done via the FLUOVIEW (FV31S-SW, Olympus, Tokyo, Japan) at a rate of approximately 5 Hz.

### 2.6 Motion and activity analysis during calcium imaging

Mouse movements were recorded at a video camera (MC-D030B, CREVIS, Korea) with infrared illumination (DR4-56R-IR85, LVS, Korea) by a custom program written in LabVIEW (National Instruments, USA) and were synchronized with two-photon imaging by a trigger generated in the program. The recorded video had 30 frames per second, with a frame size of 640 × 480 pixels. To quantify movements, the intensity difference of each pixel between frames was computed and summed across all pixels. If the accumulated value of a frame surpassed a threshold set by a blinded experimenter, the frame was categorized as movement-positive.

### 2.7 Preprocessing of calcium imaging data

#### 2.7.1 Motion correction and detection of regions-of-interests

Motion within the imaging data was rectified employing the Turboreg algorithm (Biomedical Imaging Group, Swiss Federal Institute of Technology, Lausanne, Switzerland). The CNMF-E algorithm (Zhou et al., 2018) was employed to identify regions-of-interests (ROIs), which were subsequently examined manually. Spatial ROI details were transferred to ImageJ, with the mean fluorescence for each ROI computed relative to each frame.

#### 2.7.2 Denoising and normalization

Prior to applying normalization methods, raw fluorescence signals were filtered with a Gaussian window of size 29 to mitigate noise. Subsequently, both delta F and Z-score normalization methods were applied to the data. For each ROI within a single recording session, baseline fluorescence was defined as the 30th percentile of the corresponding signal.

Delta F defined as follows:


$$\begin{array}{l} F\left(x\right)=\left(X-\mu \right)/\mu \end{array}$$

and Z score defined as follows:


$$\begin{array}{l} F\left(x\right)=\left(X-\mu \right)/\sigma \end{array}$$

where m and s indicate mean and standard deviation of baseline signals.

### 2.8 Application of machine learning

#### 2.8.1 Manual feature extraction

Handcrafted feature extraction defined as follows:


$$D=\left(\frac{X_{i}}{\sum_{i}^{n}X_{i}}\right)A-\left(\frac{X_{i}}{\sum_{i}^{n}X_{i}}\right)B$$

For ROI i in a total of n ROIs, the delta F or Z score signals X are calculated for both session A and session B. Baseline activity is defined using either session A or session B depending on the evaluation target. The difference in calcium activity (D) between matched ROIs in sessions A and B is then calculated, resulting in a vector of size n. Ratios are calculated separately for values of D > 0.3 and smaller than −0.2, each serving as a distinct feature. The value of X depends on the chosen normalization method (delta F or Z score) and the presence of movement (movement, stationary, or total). As each X calculation method generates two features (up-regulated and down-regulated), this results in a total of 12 features. Additionally, the average pairwise correlation between each ROI within a single session is incorporated as the final feature, bringing the total number of features to 13.

#### 2.8.2 Dimensional reduction, class labeling, and cross-validation

For principal component analysis (PCA), the scikit-learn library condensed input data dimensions from 13 to 6. All six principal components were utilized. Baseline, sham, and vehicle control data were labeled as non-pain; CFA, PSL, oxaliplatin, and MPTP-induced pain data as pain. Analgesics and analgesic candidates were allocated as the third class. Leave-one-subject-out (LOSO) was adopted for cross-validation (CV). Data from one mouse was reserved for testing, while the remaining data was used for training.

#### 2.8.3 False labeling management

To eliminate false labels, a classification model was trained with all data, followed by recursive testing. Data labeled as pain, within the bottom 10% for pain estimation, were deemed mislabeled and removed for further training.

### 2.9 Statistical methods

Analyses were conducted using GraphPad Prism 8 (GraphPad Software, Inc.) and Python (utilizing the scipy library). All data were presented as mean ± standard error mean (SEM). Statistical tests were performed with an unpaired *t*-test or Mann-Whitney test as appropriate. The statistical techniques used to generate each figure are described in the figure legend.

## 3 Results

### 3.1 Establishment of various types of spontaneous pain mouse models

**Figure 1:**
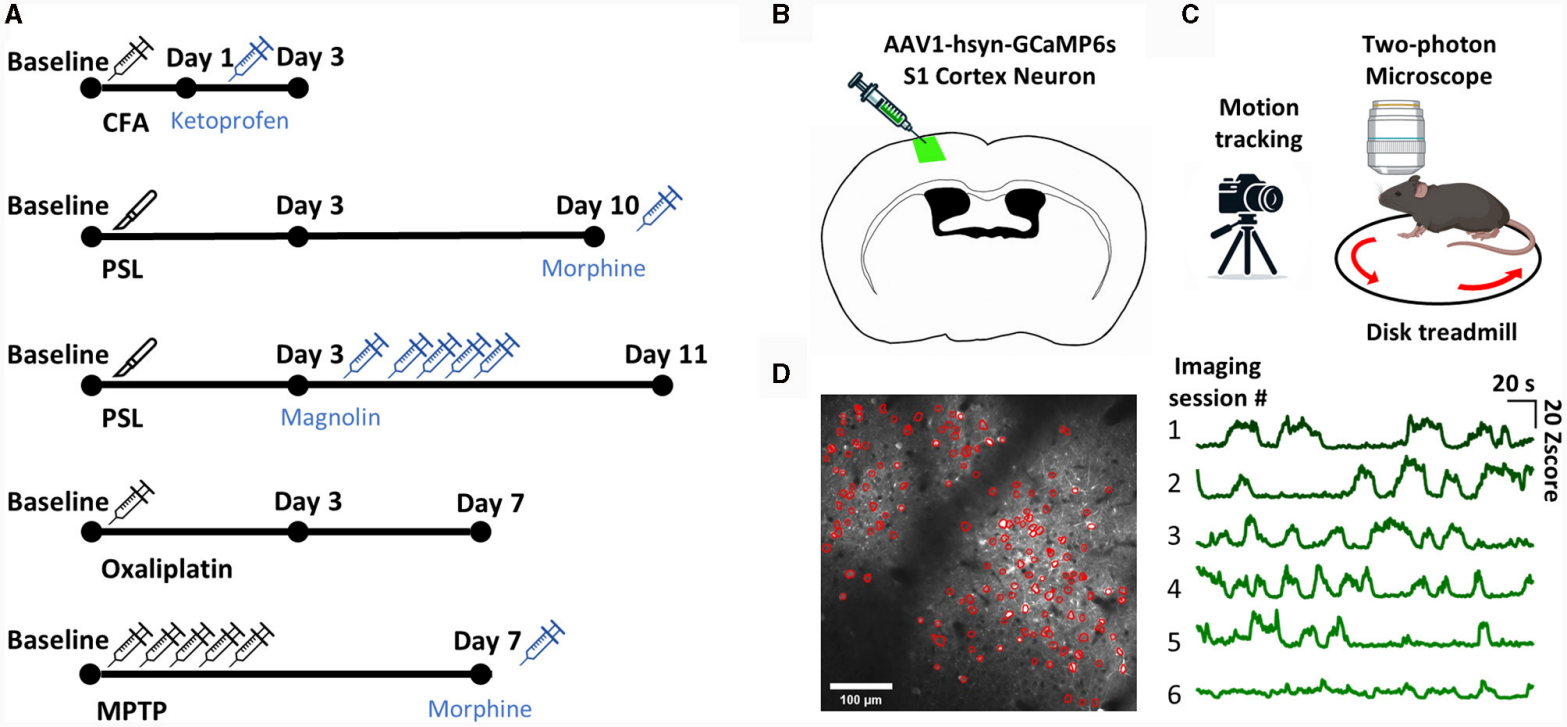
The experimental scheme. **(A)** The experimental scheme for each model. **(B)** Diagram of injection procedure for AAV-hsyn-GCaMP6s virus in S1 cortex neuron. **(C)** Diagram of the two-photon calcium imaging set-up. The head-fixed mouse was placed on a treadmill, and the left S1 neuron of the mouse was imaged with a two-photon microscope while simultaneously being recorded with a camera. **(D)** Representative image of S1 neuron ROI detection using the CNMF-E algorithm **(left)**. Example of an average calcium traces in total ROIs from each imaging session **(right)**.

We established representative inflammatory, chemical, neuropathic, and PD pain models to ensure that our ML model could cover a wide range of spontaneous pain models. We administered drugs or conducted surgery according to the protocols proposed for the pain models (Figure 1A). Subsequently, to verify the successful establishment of the pain models and the effectiveness of the administered analgesic dosages, we conducted mechanical hypersensitivity tests to assess the presence of evoked pain (Supplementary Figure 1). We then performed two-photon calcium imaging to determine the neural activity patterns of spontaneous pain. To express a calcium indicator for two-photon imaging, surgery was performed to inject GCaMP6s into the S1 of mice (Figure 1B). Afterwards, we placed the mice on a treadmill with their heads immobilized and recorded the calcium activity of layer 2/3 neurons in the left S1 of the mice while recording their movements with an infrared camera (Figure 1C). We detected the ROI of the video data using the CNMF-E algorithm and manually inspected additional ROIs (Figure 1D).

### 3.2 Manual feature extraction from S1 neuronal activity

**Figure 2:**
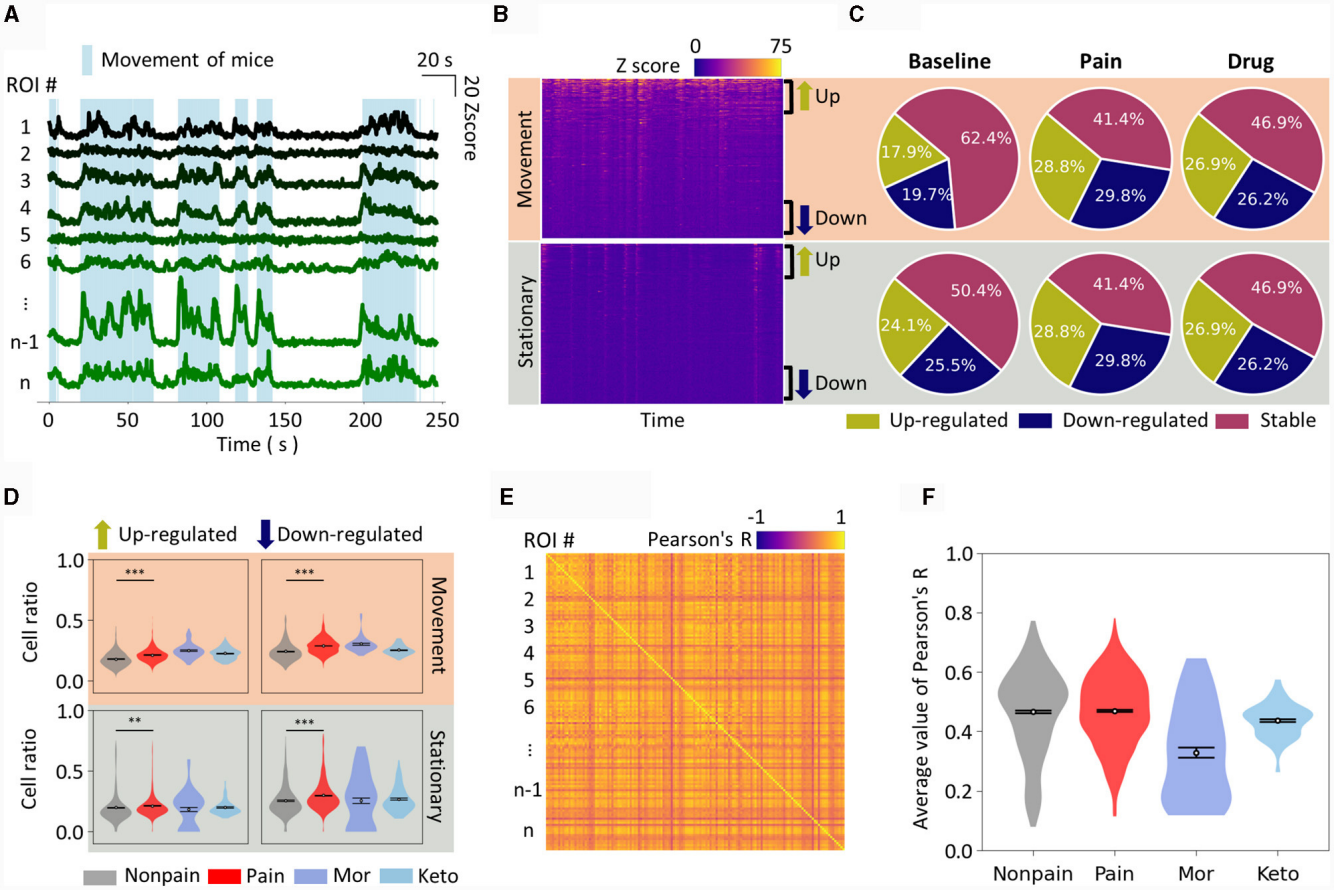
Manual feature extraction from calcium traces. **(A)** Plot of calcium traces of individual ROIs during an approximately 4 min imaging session. Only 8 representative ROIs are displayed. The light blue shade indicates the periods when mice have movement. The scale bar for the graph is displayed in the top right corner. **(B)** Calcium traces of all ROIs are displayed as a heatmap, distinguishing between movement and stationary periods. The ROIs are sorted in descending order of activity level. Therefore, the top part of each heatmap corresponds to up-regulated neurons from the baseline, while the bottom part corresponds to down-regulated neurons. **(C)** Pie charts show the cell ratio of up-regulated, down-regulated, and stable neurons in non-pain baseline, pain, and drug-induced analgesic states. The top represents analysis during movement, and the bottom during stationary states. **(D)** Similar to **(C)**, the cell ratio in each state is displayed as a violin plot. Drug-induced analgesic states are plotted separately for morphine and ketoprofen. **(E)** An example of correlation analysis is represented as a heatmap. The intersection of pairs of ROIs is marked with their correlation coefficient. **(F)** The average correlation coefficient of all ROI pairs is calculated as the correlation feature value for a single session. The correlation feature values for each state are presented as violin plots. The data are presented the mean ± SEM; N.S., not significant; **P < 0.01; ***P < 0.001 as determined by an unpaired *t*-test.

After obtaining calcium signals from various pain models, we initially focused on extracting key features from these S1 signals to determine the presence of spontaneous pain. Several studies have reported changes in S1 neuronal activity in chronic pain conditions, notably an increase in excitatory neuronal activity (Kei et al., 2011; Eto et al., 2012; Cichon et al., 2017). Importantly, under our experimental conditions, the mice exhibited random movements, and their neuronal activity was distinctly different when moving compared to when stationary, as shown in Figure 2A. Therefore, we hypothesized that the differences in neuronal activity during movement vs. stationary states could contain distinct and informative patterns.

Therefore, we first arranged each neuron, that is, the ROI, by comparing their Z-scores to the baseline and sorted them from the most up-regulated to the most down-regulated. When separated into movement (Figure 2B, top) and stationary states (Figure 2B, bottom) and displayed as heatmaps, the up- and down-regulated ROIs were distinctly visible. We set a uniform threshold across all data, defining neurons as up-regulated, down-regulated, or stable if they exceeded this predetermined threshold (see methods for details). We then depicted the dynamics of neuronal activity during baseline, pain, and drug-induced analgesic conditions, displaying the results for both movement (Figure 2C, top) and stationary states (Figure 2C, bottom) in separate. In the pain state, both up- and down-regulated neuronal activity ratios were increased when the mice were walking on the treadmill (Figure 2D, top). The neuronal activity in the S1 during drug-induced analgesic states, particularly with morphine and ketoprofen, was noteworthy for its distinctive patterns. Notably, the S1 neuronal activity pattern in the drug-induced analgesic state either resembled or even intensified compared to the pain state (Figure 2D, top). For instance, morphine administration resulted in higher up-regulated neuronal activity ratio. On the other hand, when the mice were stationary, up-regulated neuronal activity ratios did not show a statistically significant difference (Figure 2D, bottom left). Conversely, down-regulated neuronal activity ratios increased in the pain state and returned to baseline in the drug-induced analgesic state (Figure 2D, bottom right). In addition, we calculated the Pearson correlation over time between ROIs within an imaging session. When plotted as a heatmap (Figure 2E), various correlation coefficients emerged among the ROIs. We averaged the correlation values across all ROIs to define the correlation feature of that particular imaging session. We observed that the level of correlation feature among individual cells was similar in non-pain and pain states, but a marked difference was evident in the drug-induced analgesic state (Figure 2F).

Many of the aforementioned metrics showed distinct differences in pain, non-pain, and drug-induced analgesic states, whether this metric is adequate for classification needs to be considered. For instance, while there was a statistically significant difference in features between groups (Figure 2D), the area under curve metrics (AUC) for distinguishing pain from non-pain at the level of individual imaging sessions were not as robust as the statistical significance (Table 1). For example, the down-regulated cell ratio during movement, normalized by delta F, which shows the highest discrimination ability, distinguishes between pain states and both baseline and drug-induced analgesic states with an AUC performance of 0.68. This can be considered a fairly decent performance; however, it still fails to apply to certain drugs. As can be seen in the top right graph of Figure 2D, morphine does not get distinguished from pain.

**Table 1:** Summary of pain classification performance of features.

**Feature #**	**Normalization method**	**Neuronal dynamics**	**Movement state**	**Baseline vs. pain**	**Pain vs. analgesics**	**Pain vs. analgesics + baseline**
1	Z score	Up	Movement	0.63	0.63	0.58
2			Stationary	0.58	0.56	0.58
3			Total	0.64	0.59	0.59
4		Down	Movement	0.67	0.67	0.67
5			Stationary	0.63	0.60	0.63
6			Total	0.65	0.58	0.63
7	delta F	Up	Movement	0.68	0.62	0.61
8			Stationary	0.62	0.52	0.60
9			Total	0.68	0.58	0.62
10		Down	Movement	0.71	0.59	0.68
11			Stationary	0.63	0.57	0.61
12			Total	0.69	0.52	0.65
13	Correlation			0.51	0.65	0.52

These results imply that while simple features of neuronal activity hold statistical significance between groups, their reliability at the AUC metric level for pain and analgesic effects detection is relatively low. Moreover, the state of analgesia induced by drugs does not represent a non-pain state but rather exhibits distinct patterns specific to each analgesic.

### 3.3 Machine learning approach for pain classification

Manually extracted features showed statistically significant differences across non-pain, pain, and drug-induced analgesic states. However, we found that the S1 patterns in drug-induced analgesic state did not simply return to the normal non-pain state, but instead exhibited a unique pattern. Consequently, at the level of each manually extracted feature, it was not possible to distinctly classify the pain state, with non-pain and drug-induced analgesic states often grouped together. To address this issue, in this study, we propose a novel three-class classification model that considers the drug-induced analgesic state as a third class, instead of a simple binary classification model. This is crucial as it prevents the drug-induced analgesic state from being misinterpreted as pain states, by distinctly identifying the unique neural patterns seen under the influence of analgesic drugs.

**Figure 3:**
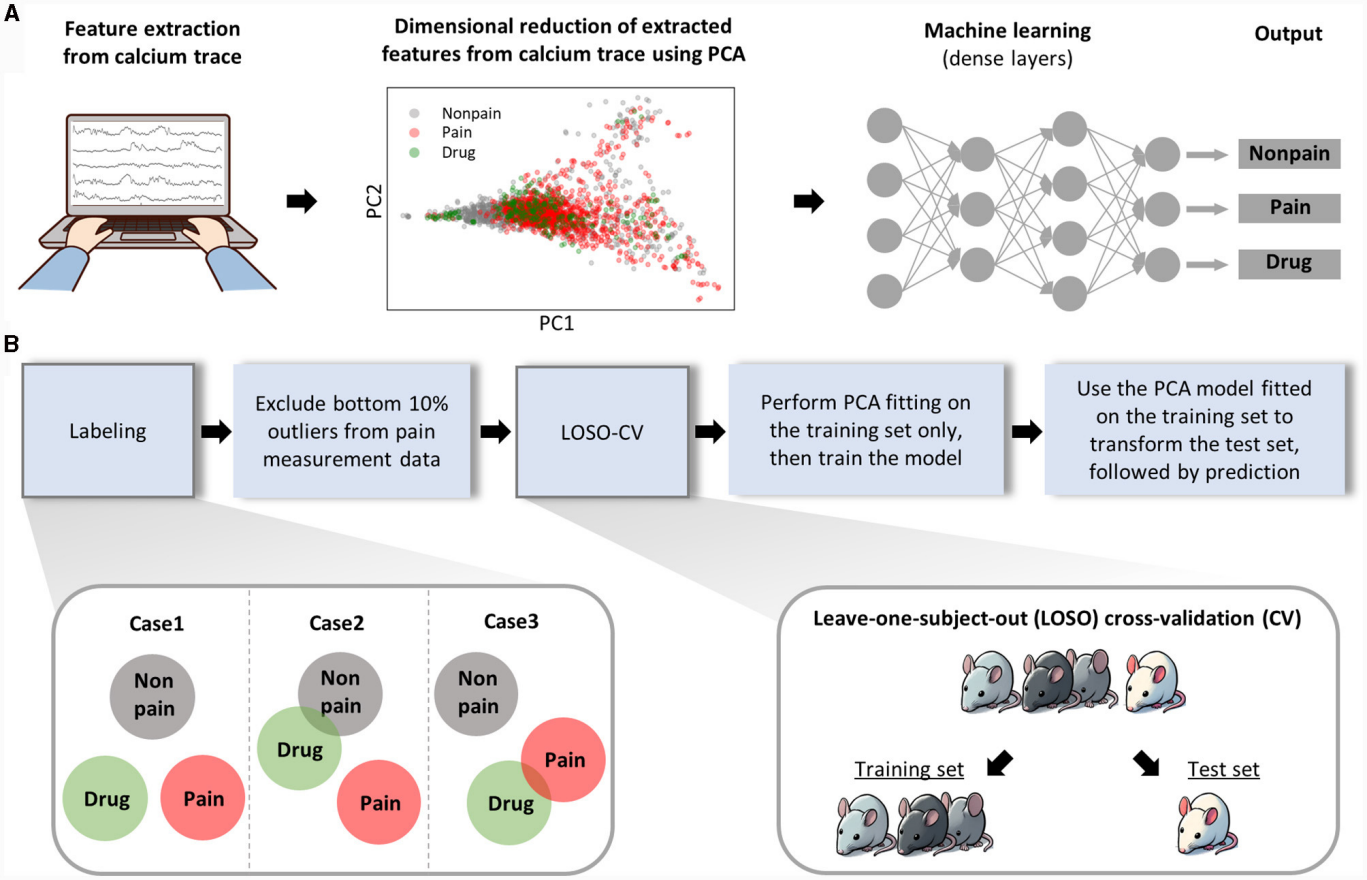
Analytical workflow of the machine learning analysis. **(A)** This part represents the analysis procedure from raw data to pain prediction. Features are manually extracted from calcium traces. Thirteen manually extracted features were reduced to six through PCA, and the first and second principal components were used to create a 2D scatter plot. The diagram simplifies the structure of the machine learning model. Detailed information on the architecture of ML model is provided separately in the methods section. **(B)** The procedure for training and evaluating the machine learning model is sequentially presented.

As mentioned above, we extracted features for pain assessment from the dynamics of neural activity relative to the baseline and from the correlation characteristics between activities of each neuron (Figure 3A, left). To minimize redundancy among the total 13 features (see methods for details) and prevent overfitting in the ML model, we performed PCA, consequently narrowing down the number of features to six. When we plotted the data of each class using the top two principal components in a two-dimensional scatter plot (Figure 3A, middle), we found it still challenging to clearly distinguish each class. Consequently, we put these data into a ML model composed of dense layers to train the model to differentiate each class (Figure 3A, right).

The drug-induced analgesic state was considered a separate class from non-pain class as well as pain class. The separability of the drug class from other classes can be anticipated in various scenarios (Figure 3B, left bottom). Next, considering the nature of spontaneous pain data, we could guarantee that pain was present during our recording of neuronal activity, which might lead to false labeling. To exclude this false labeling, we initially trained the ML model with all the data and recursively retested the training set data. We regarded the bottom 10 percent of data, which was least classified as pain, as false labels and removed them from the training set. For model validation, we employed a Leave-One-Subject-Out Cross-Validation (LOSO-CV) approach, where all data obtained from a single mouse was treated as the test set (Figure 3B, right, bottom). Lastly, we ensured the separation of the test set information during the PCA fitting and model training processes to maintain the integrity of our validation process.

### 3.4 Validation of a spontaneous pain detection by the suggested ML model

**Figure 4:**
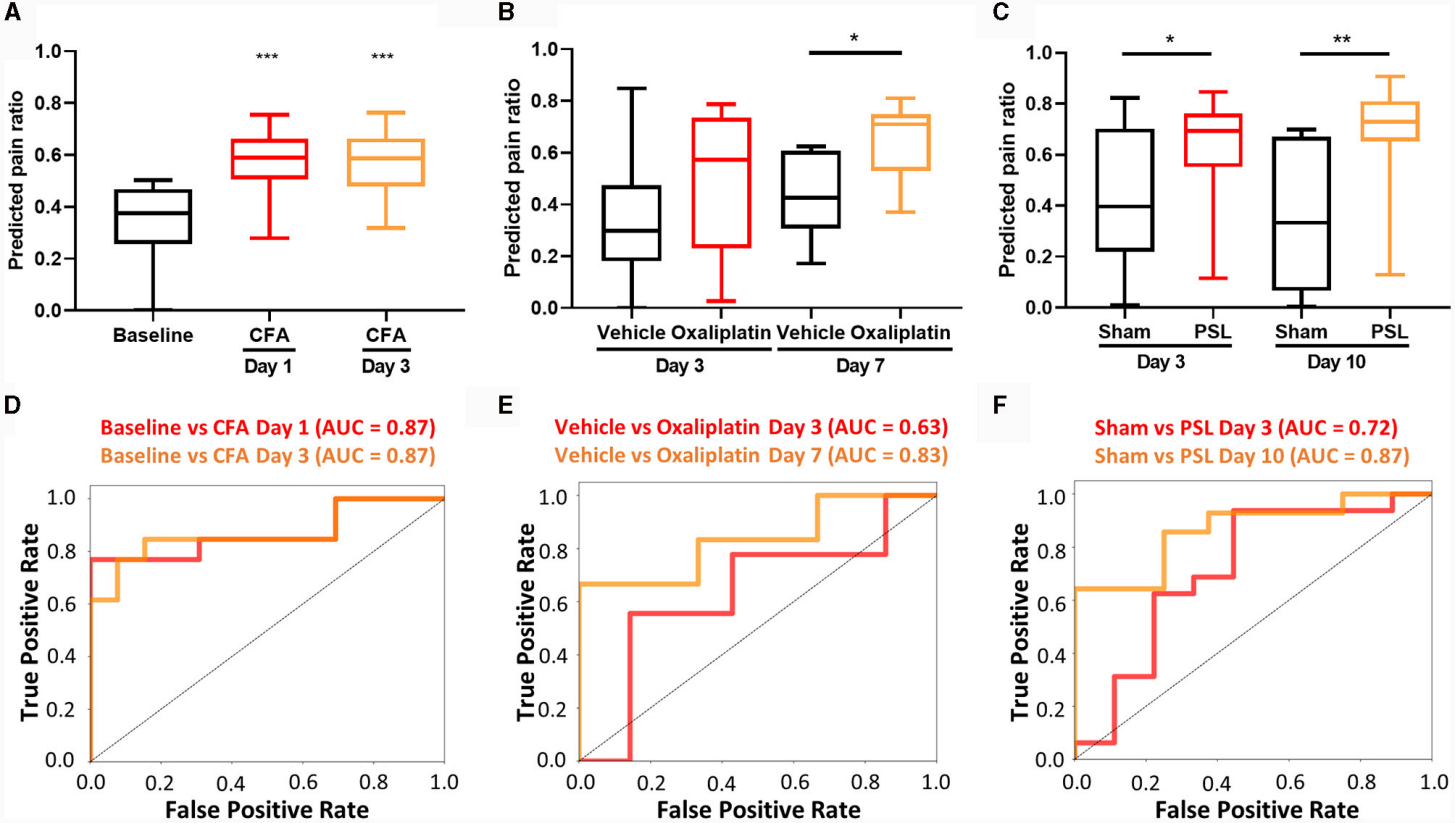
Detection of spontaneous pain for each pain model. The predicted pain value of **(A)** CFA (10 μl, s.c.) model (*n* = 13 per group), **(B)** oxaliplatin (6 mg/kg, i.p.) model (*n* = 7 for vehicle group of day 3, *n* = 9 for oxaliplatin group of day 3, and *n* = 6 for both group of day 7), and **(C)** PSL model (*n* = 9 for sham group of day 3, *n* = 16 for PSL group of day 3, *n* = 8 for sham group of day 10, and *n* = 16 for PSL group of day 10). Classification performance in pain condition of **(D)** CFA model, **(E)** oxaliplatin model, and **(F)** PSL model. The data are presented the mean ± SEM; *P < 0.05, **P < 0.01, ***P < 0.001 by unpaired *t*-test.

To assess the effectiveness of our three-class ML classification method, we first determined if non-pain and pain states could be distinctly identified from one another. The results showed that our ML model was able to significantly distinguish between non-pain and drug-induced analgesic state from the pain state. In the CFA model, distinct S1 neural activity patterns were detected on both day 1 and day 3 following CFA injection into the hind paw (Figure 4A). On the other hand, in the oxaliplatin model following intraperitoneal injection, slight differences in S1 neural activity patterns were detected on day 3, differing from those reported in evoked pain (Li et al., 2015). By day 7, marked differences in activity patterns were detected (Figure 4B). These findings suggest that spontaneous pain induced by oxaliplatin may have a slower onset compared to evoked pain. In the PSL model, which is one of the models for neuropathic pain, significant differences in neural activity patterns were observed on both day 3 and day 10 (Figure 4C). As a result of verifying the classification performance for non-pain and pain states, all data except for day 3 of oxaliplatin showed a high level of performance (Figures 4D–F).

**Figure 5:**
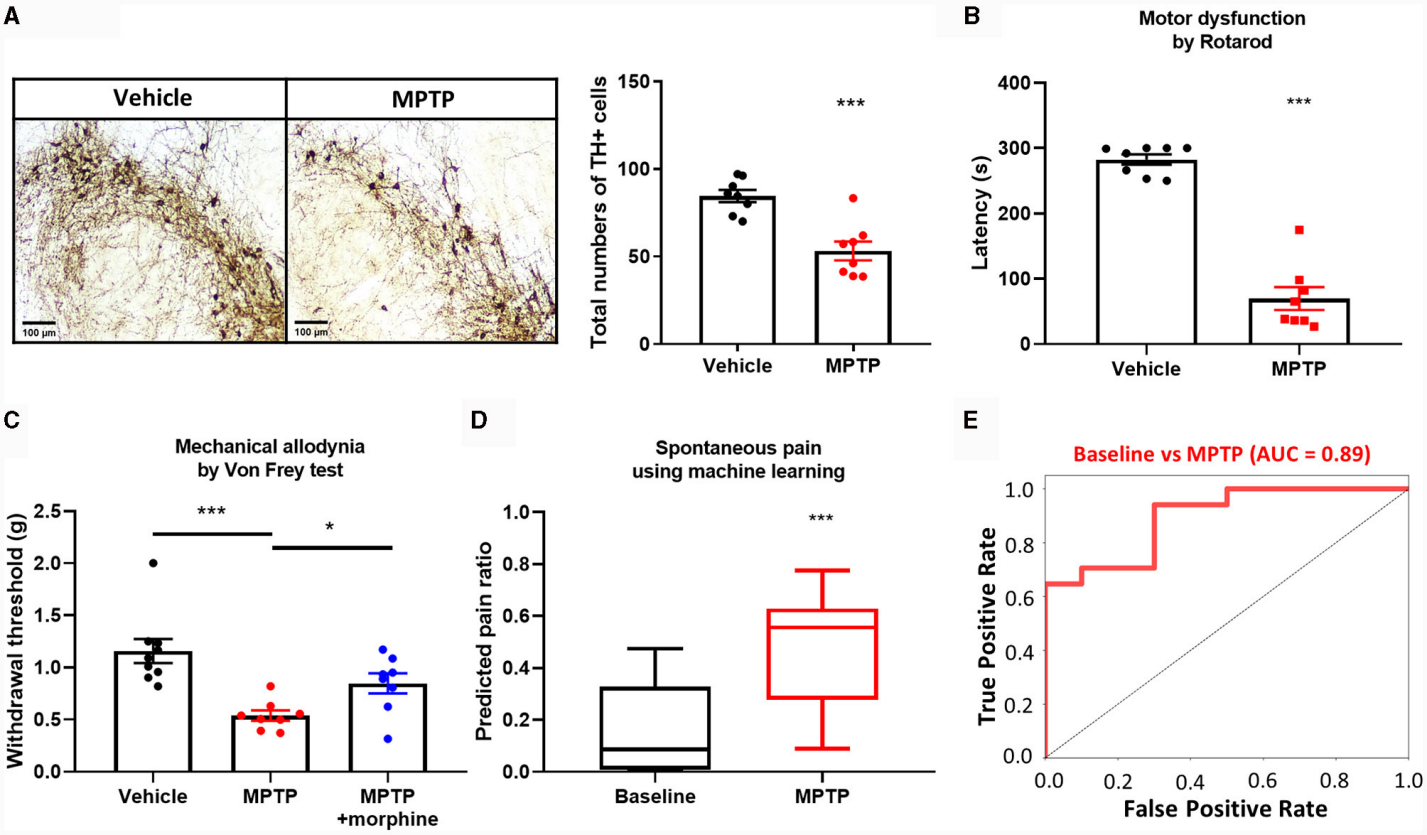
Establishment of MPTP-induced PD model and detection of spontaneous pain. **(A)** Representative images and quantification of TH+ cells reduction in SNc of MPTP (30 mg/kg, i.p.) -induced PD model (*n* = 8 per group). **(B)** Reduced motor function (*n* = 8 per group), and **(C)** increased mechanical allodynia response and morphine (5 mg/kg, i.p.) -induced analgesic effect in the PD model (*n* = 9 for vehicle group, *n* = 8 for MPTP and MPTP + morphine group). **(D)** The predicted pain value of MPTP-induced PD model in pain condition (*n* = 10 for baseline group and *n* = 17 for MPTP group). **(E)** Classification performance in pain condition of MPTP-induced PD model. The data are presented the mean ± SEM; *P < 0.05, ***P < 0.001 by unpaired *t*-test.

We tried to apply this ML method to evaluate spontaneous pain in PD model. It is known that patients with PD have a very poor quality of life due to pain, but no preclinical studies have measured spontaneous pain in PD (Kuopio et al., 2000; Buhidma et al., 2020). It is also difficult to measure with CPP because the reward system can be impaired (Huston et al., 2013). Therefore, we tried to use ML methods to measure spontaneous pain in PD. First, we identified the PD model by injecting the neurotoxin MPTP, which resulted in a decrease in dopaminergic neurons located in the substantia nigra and a decrease in motor function (Figures 5A, B). When we measured the spontaneous pain of the PD model using ML methods, we found that the pain state was clearly distinguished from baseline, and the PD model injected with morphine also showed pain levels similar to baseline (Figure 5C). We observed significant differences in neural activity patterns when spontaneous pain was judged by ML techniques in a PD model (Figures 5D, E), suggesting that ML analysis methods can be used to determine spontaneous pain in PD, and that spontaneous pain exists in PD.

### 3.5 Validation and application of the ML model in the efficacy assessment of analgesics

**Figure 6:**
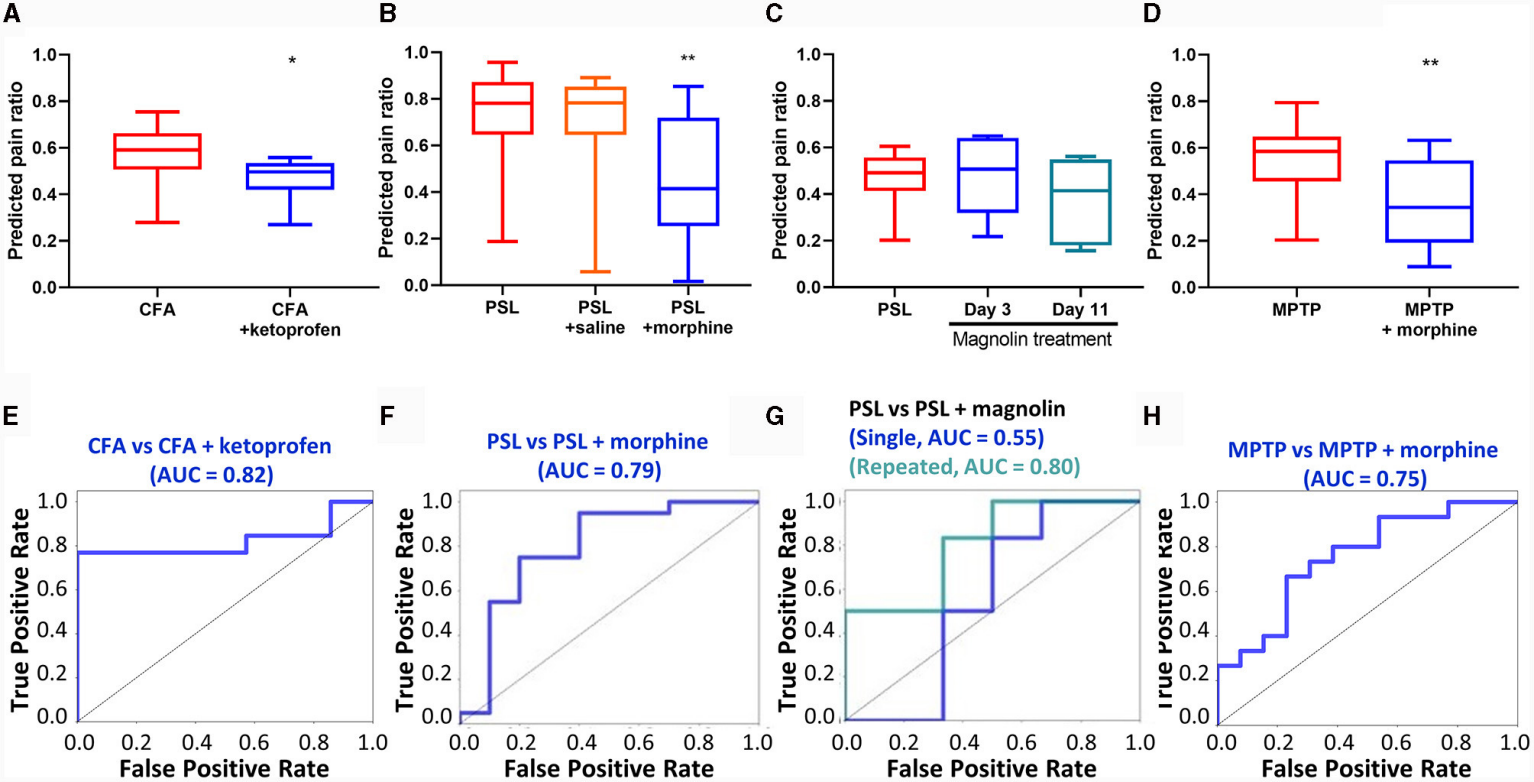
Detection of analgesic effect of spontaneous pain in each pain model. The predicted pain value of **(A)** CFA model injected with ketoprofen (*n* = 13 for CFA group and *n* = 7 for CFA + ketoprofen group), **(B)** PSL model received saline or morphine via intraperitoneal (i.p.) injection (*n* = 11 per group), **(C)** PSL model i.p. injected with magnolin (*n* = 7 per group), and **(D)** MPTP-induced PD model i.p. injected with morphine (*n* = 16 for MPTP group and *n* = 14 for MPTP + morphine group). Classification performance under analgesic conditions for **(E)** CFA + ketoprofen model, **(F)** PSL + morphine model, **(G)** PSL + magnolin, and **(H)** MPTP + morphine model. CFA model data are presented the mean ± SEM; N.S., not significant; *P < 0.05, **P < 0.01 by Mann-Whitney test. The remaining data are presented as unpaired *t*-test.

We also used ML classification models to determine the effectiveness of analgesics in each pain model. Ketoprofen, an anti-inflammatory drug, was used to treat inflammatory pain caused by CFA injections. Similarly, morphine, an opioid drug commonly used in clinical practice, was used for managing neuropathic pain induced by PSL (Cooper et al., 2017). Significant changes in neuronal activity patterns were observed following intraperitoneal injections in two models. In the CFA model, these changes were detected after administering ketoprofen, as shown in Figure 6A. Similarly, in the PSL model, notable alterations in neural activity were observed following the administration of morphine, illustrated in Figure 6B.

Next, we employed ML analysis to evaluate the analgesic effects of magnolin. Magnolin is a major component of the magnolia family and is known to have anti-inflammatory, antibacterial, and anti-allergic effects (Chae et al., 1998; Kim et al., 1999; Wang et al., 2019). Among these effects, inhibition of ERK/RSK2 signaling, which is involved in the chronification of pain, suggests that magnolin has potential as an analgesic (Kondo and Shibuta, 2020). We analyzed neuronal activity using our ML model after both single and repeated administrations of magnolin in the PSL model (Figure 6C). However, our findings indicate that magnolin, under the current administration protocols, does not effectively attenuate spontaneous pain in the PSL model.

Moreover, significant differences in neural activity patterns were observed after intraperitoneal injection of morphine in the PD model. These results suggest that morphine is effective for providing analgesia in the PD model (Figure 6D). Our analysis of the classification performance for pain and drug states demonstrated excellent results across all datasets, with the notable exception of datasets involving single administrations of magnolin, as depicted in Figures 6E–H. The difficulty in differentiating pain states following single administrations of magnolin is speculated to be due to its low analgesic efficacy.

## 4 Discussion

In this study, we developed an ML algorithm to detect spontaneous pain in animals. The algorithm uses calcium activity data from S1 neurons recorded using two-photon microscopy. We extracted information on the relative up and down-regulated ratio of up and down-regulated cells in calcium activity and the correlation level of activity between cells as features for input data for the ML model. The proposed ML model reliably classified spontaneous pain and analgesic efficacies in mouse models of inflammatory and neuropathic pain. We also demonstrated that a mouse model of PD induced by MPTP injection exhibits spontaneous pain, which showed different dynamics from evoked pain. These results indicate that the suggested ML algorithm is a novel tool for measuring spontaneous pain in animals and can contribute to the development of clinically relevant pain treatments in preclinical settings.

The measurement of spontaneous pain in the preclinical level of analgesic development is critical for translation to clinical applications (Bushnell et al., 2013). Due to this demand, behavioral assays for measuring spontaneous pain, such as the CPP and the GS, have been developed. Each of these methods based on animal behaviors has limitations. For instance, CPP (King et al., 2009) requires learning and memory capabilities, as well as an intact reward system, complicating its use in pain research within models of Alzheimer's or PD (Huston et al., 2013). In contrast, the method we propose does not have these dependencies since it utilizes brain signals. As an example, we confirmed for the first time the presence of spontaneous pain in a mouse model of PD and verified the analgesic effects of morphine in this pain model (Figure 5D). The applicability of the method proposed in this study extends beyond the confines of traditional spontaneous pain measurement techniques, offering a versatile tool that promises to advance research across a broader spectrum of pain models. In addition, the spontaneous pain measurement technique proposed in this study can be used in conjunction with existing methods, allowing for mutual complementarity.

To detect spontaneous pain through brain signal observation, we selected the S1 cortex for its integral role in pain processing, acting as a crucial hub that processes nociceptive information. Pain signals reach the S1 cortex through several pathways: directly from the spinal cord (Cai et al., 2023), from the spinal cord via the thalamus (Basbaum et al., 2009), and through the spino-parabrachial-thalamic route (Krout and Loewy, 2000; Deng et al., 2020; Bak et al., 2021; Li et al., 2023) and relays these signals to the anterior cingulate cortex (Singh et al., 2020). Across various chronic pain models, which are commonly associated with the occurrence of spontaneous pain, the S1 cortex has demonstrated a critical role in pain processing. In the nerve injury model, alterations in neural activity were observed to vary according to the subtype. Pyramidal neurons exhibited increased neural activity, while local inhibitory interneurons were regulated in a direction that overall increased net activity, depending on the specific interneuron subtype (Cichon et al., 2017). Similar patterns are seen in the CFA pain model, with increased synchronized neuronal activity and connectivity evident within the S1 cortex (Okada et al., 2021). In oxaliplatin-induced neuropathic pain, the down-regulation of Kv2.2 potassium channels in the somatosensory cortex correlates with increased neuronal and cortical excitability (Thibault et al., 2012). These findings across different models align with recent research, indicating the importance of S1 activity in detecting spontaneous pain.

In previous research, our team developed an algorithm that uses DL to detect pain from S1 calcium signals (Yoon et al., 2022). In the current study, we introduced an ML model that, while aiming to achieve similar goals as our prior research, offers a range of distinct benefits. Firstly, as a black box model, the previous DL model of ours could not provide information about which features from the calcium signal contribute to pain detection. This lack of interpretable explanation makes it difficult to identify malfunctions in the model and does not offer insights into the modulation of neurons for pain alleviation. In contrast, an ML model that uses interpretable manually extracted features can easily improve performance by modifying or adding features, and it provides physiological information about pain representation in S1 neurons. Finally, from a general perspective, ML as a shallower model compared to DL has a lower risk of overfitting and its simplicity and faster data processing speed allow it to be embedded in small mobile devices for fast and real-time pain detection performance (Petrosky et al., 2018; Salman and Liu, 2019).

An approach utilizing ML for assessing spontaneous pain is notable not just for its ability to report the presence or absence of pain. It also enables the observation of brain activity patterns during both pain episodes and periods of drug-induced analgesia. Particularly in this study, by utilizing manual feature extraction in conjunction with ML techniques, we observed brain signal patterns that can be intuitively understood by humans, unlike those derived from DL models. Previous studies reported that neuronal activity of the S1 was increased in chronic pain (Kim S. K. et al., 2016; Ishikawa et al., 2018; Bak et al., 2021), and diverse modulation of inhibitory interneurons contribute to hyperactivity in pyramidal neurons (Cichon et al., 2017). The activity patterns in pain states in the current study aligned with these previous findings. However, our analysis also revealed the novel aspects that had not previously been reported. We found that S1 neurons had different patterns based on the interplay between upregulated and downregulated neuronal activity, as well as between active movement and stationary states. Interestingly, it was observed that when the mice remained stationary, neuronal activity under the influence of both ketoprofen and morphine reverted to normal levels. In contrast, during movement, neuronal activity under each drug exhibited a distinct pattern, not simply returning to the baseline. Furthermore, the analysis highlighted the correlation between cells, marking it as a newly identified and potent feature for differentiating the analgesic effects of various drugs. These results enhance our understanding of the complexities involved in pain management and the effectiveness of analgesic drugs, particularly in relation to movement and stationary states, offering new perspectives for future research in this field.

In PD, pain symptoms often remain underestimated although they significantly reduce the quality of life in patients (Defazio et al., 2008; Beiske et al., 2009). Despite the significant impact that pain can have on PD patients, preclinical research on PD pain has been limited due to the difficulty of measuring spontaneous pain in animal models of PD (Buhidma et al., 2020). For example, PD exhibits disruption of the dopaminergic reward system (Kapogiannis et al., 2011), which makes it difficult to apply CPP, a common preclinical pain assessment method. In this study, we propose a ML-based method for measuring spontaneous pain in PD models. Our model was able to reliably detect spontaneous pain in MPTP-injected PD mouse models, and it also confirmed the analgesic effect of morphine. We believe that our proposed model can provide a valuable tool for preclinical research on PD pain. By enabling more accurate and reliable measurement of spontaneous pain, our model can help to advance our understanding of the causes and symptoms of pain in PD models, accelerate the development of new pain therapies, and improve the quality of life for PD patients.

The study of magnolin in pain management is noteworthy due to its unique targeting of ERK signaling, a pathway known for its role in pain modulation (Kondo and Shibuta, 2020). Magnolin is known to target the ERKs/RSK2 signaling pathway (Lee et al., 2015), inhibiting cell migration and invasion, and has been suggested as a biological target in pain management (Giraud et al., 2021). In this study, magnolin treatment initially did not show a statistically significant difference in analgesic efficacy compared to the control group, unlike morphine and ketoprofen. However, after repeated doses twice daily for 4 days, a significant analgesic effect was observed (Figure 6C). The results observed following the initial injection of magnolin suggest a discrepancy in its analgesic effectiveness between evoked and spontaneous pain (Cao et al., 2012; Kim N. et al., 2023). These results lead us to suggest that it would be more appropriate to test repeated dosing of magnolin in clinical trials. While there are no clinical trial results yet for the analgesic effects of magnolin, this case study demonstrates the ability of the ML model to assess analgesic effects on spontaneous pain of a candidate drug to various drug dosages. This information can be used to optimize drug dosage for clinical trials.

The use of ML to analyze brain signals enables the detection of spontaneous pain and analgesic effects that have been difficult to detect with conventional analysis methods alone. However, there might be skepticism about whether the classification by ML truly detects spontaneous pain or merely identifies other artifacts. This limitation arises from the fact that chronic pain models exhibit not only pain but also various other differences when compared to control groups, and that S1 brain signals are involved in a wide range of functions beyond just pain (Roudaut et al., 2012; Abraira and Ginty, 2013; Kim et al., 2017). Despite potential skepticism, our study demonstrates the sufficient utility of ML classifications in achieving the intended objectives. This conclusion is based on three key aspects. Firstly, S1 neurons display distinct patterns in chronic pain situations, as evidenced by various studies demonstrating critical role of the S1 in pain sensation and processing across different species, including humans (Omori et al., 2013; Kim et al., 2017; Bak et al., 2021). Secondly, the use of diverse pain models in our training set increases the likelihood of detecting a common pain pattern, suggesting that our ML model is recognizing a consistent pain signature rather than model-specific anomalies. Lastly, the primary purpose of our research was not solely to capture the pain itself, but rather to effectively classify different pain models and analgesic responses. In this study, we have conducted extensive validation of our ML model using a variety of pain models and analgesics, highlighting its practical relevance and adequacy in meeting the objectives of preclinical analgesic screening.

## 5 Summary

We have developed an ML model to more precisely measure spontaneous pain and analgesic effect in mice, utilizing calcium signals in the S1 region. This ML model employs manually extracted features, such as dynamic variations in calcium levels and inter-cellular activity correlations, to categorize data into three distinct pain states: non-pain, pain, and drug-induced states. Our extensive testing across various pain models and medications demonstrates the heightened accuracy of the ML model in detecting both pain and analgesic effects. This ML model addresses the interpretability and analysis challenges inherent in the previous DL model. Its improved capabilities mark a significant step forward in expediting the development of novel therapies, thereby advancing the field of pain research.

## Data availability statement

The original contributions presented in the study are included in the article/Supplementary material, further inquiries can be directed to the corresponding author.

## Ethics statement

The animal study was approved by Kyung Hee University Institutional Animal Care and Use Committee. The study was conducted in accordance with the local legislation and institutional requirements.

## Author contributions

MSB: Conceptualization, Data curation, Formal analysis, Investigation, Project administration, Validation, Visualization, Writing – original draft. HP: Conceptualization, Data curation, Investigation, Methodology, Validation, Visualization, Writing – original draft. HY: Methodology, Project administration, Validation, Writing – review & editing. GC: Funding acquisition, Writing – review & editing. HS: Investigation, Writing – review & editing. SS: Investigation, Writing – review & editing. TK: Resources, Writing – review & editing. KL: Resources, Writing – review & editing. UN: Writing – review & editing. SJK: Funding acquisition, Supervision, Writing – review & editing. SKK: Conceptualization, Funding acquisition, Project administration, Supervision, Writing – review & editing.
