# Supplementary material for: Machine learning-based evaluation of spontaneous pain and analgesics from cellular calcium signals in the mouse primary somatosensory cortex using explainable features
Source: Front Mol Neurosci. 2024 Feb 21;17:1356453. doi: 10.3389/fnmol.2024.1356453 (PMC10915002; doi:10.3389/fnmol.2024.1356453)
Supplement: Supplementary file 1 [file Data_Sheet_1.PDF]

Supplementary Material

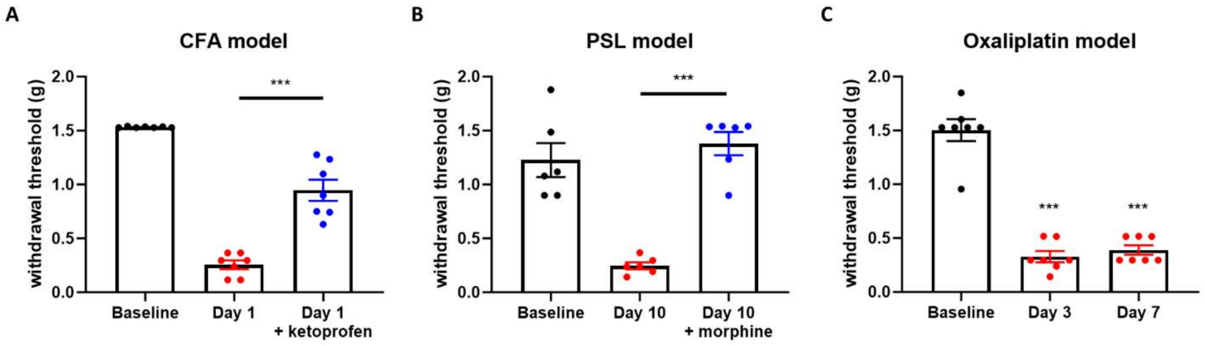

Supplementary Figure 1. Evaluation of evoked pain in CFA, PSL, and oxaliplatin models. (A) In the CFA model (n = 7), tests were conducted 1 day after CFA injection (10 μl, s.c.) into the plantar surface of the right hind paw, and increased mechanical allodynia response and analgesic effect with ketoprofen (50 mg/kg) were observed. **(B)** In the PSL model (n = 6), tests were performed 10 days after right sciatic nerve ligation surgery, demonstrating increased mechanical allodynia response and analgesic effects with morphine (5 mg/kg). **(C)** In the oxaliplatin model (n = 7), tests were carried out both 3 and 7 days after oxaliplatin injection (6 mg/kg, i.p.), indicating increased mechanical allodynia response. The data are presented the mean ± SEM; ***$P < 0.001$ by unpaired t-test.
